# Does Obesity Modify the Relationship between Exposure to Occupational Factors and Musculoskeletal Pain in Men? Results from the GAZEL Cohort Study

**DOI:** 10.1371/journal.pone.0109633

**Published:** 2014-10-17

**Authors:** Anastasia Evanoff, Erika L. Sabbath, Matthieu Carton, Sebastien Czernichow, Marie Zins, Annette Leclerc, Alexis Descatha

**Affiliations:** 1 Univ Versailles St-Quentin, Versailles, France; 2 UMS 011 Population-based Epidemiologic Cohorts Unit Inserm, Villejuif, France; 3 Harvard College, Cambridge, MA, United States of America; 4 Harvard Center for Population and Development Studies, Cambridge, MA, United States of America; 5 Department of Nutrition, Assistance Publique-Hopitaux de Paris, Ambroise Paré University Hospital, Boulogne-Billancourt, France; 6 Occupational Health Unit/EMS (Samu92), AP-HP, University hospital of Poincaré, Garches, France; CUNY, United States of America

## Abstract

**Objective:**

To analyze relationships between physical occupational exposures, post-retirement shoulder/knee pain, and obesity.

**Methods:**

9 415 male participants (aged 63–73 in 2012) from the French GAZEL cohort answered self-administered questionnaires in 2006 and 2012. Occupational exposures retrospectively assessed in 2006 included arm elevation and squatting (never, <10 years, ≥10 years). “Severe” shoulder and knee pain were defined as ≥5 on an 8-point scale. BMI was self-reported.

**Results:**

Mean BMI was 26.59 kg/m^2^ +/−3.5 in 2012. Long-term occupational exposure to arm elevation and squatting predicted severe shoulder and knee pain after retirement. Obesity (BMI≥30 kg/m^2^) was a risk factor for severe shoulder pain (adjusted OR 1.28; 95% CI 1.03, 1.90). Overweight (adjusted OR 1.71; 1.28,2.29) and obesity (adjusted OR 3.21; 1.90,5.41) were risk factors for severe knee pain. In stratified models, associations between long-term squatting and severe knee pain varied by BMI.

**Conclusion:**

Obesity plays a role in relationships between occupational exposures and musculoskeletal pain. Further prospective studies should use BMI in analyses of musculoskeletal pain and occupational factors, and continue to clarify this relationship.

## Introduction

Musculoskeletal disorders (MSDs) include a wide range of diseases and injuries that comprise the largest category of work-related illnesses. MSDs are a main cause of disability, especially in aging populations. [1] Many studies have shown that occupational factors such as repeated exposure to arm elevation or squatting in the workplace predict subsequent MSDs in the shoulders and knees. [2]–[4] Previous analyses have been performed on these joints with a particular focus on associations between long-term biomechanical exposure and incidence of severe pain; consistent associations have been found between repeated exposure to arm elevation or squatting in the workplace and severe shoulder and knee pain. [5], [6] Self-reported symptoms of pain are the most common criterion used to assess the presence of MSDs. [1] Recommendations emphasize the use of instruments such as Nordic-style questionnaires, [7] especially with numeric scales of disability intensity and pain [8].

Obesity has become a worldwide epidemic, affecting over one-third of the adult population in the United States and about 15% in France. [9] Obesity may also be a risk factor for shoulder and knee pain [10]–[13]; thus, rising obesity rates could partly explain the increasing levels of observed musculoskeletal pain and disability [14].

In addition to being a risk factor for MSDs, recent studies have found that obesity may also be a consequence of occupational exposures, potentially mediating and/or modifying effects of occupational factors on musculoskeletal pain. [15]–[17] Furthermore, occupational exposures may be risk factors for obesity. [15], [16] Some suggest that obesity may increase mechanical forces on the joints and change the metabolic demands of the body, both of which would lead to higher rates of MSDs. [13], [18] Thus, the nature of the interrelationships between occupational exposures, obesity, and musculoskeletal pain are complex; more research is needed to understand the nature of such relationships.

This study aims to disentangle associations between occupational exposures, obesity, and pain in shoulders and knees. We hypothesized that occupational exposures may be significant contributors to incidence of musculoskeletal pain among overweight and obese patients, and that the relationships may differ for upper and lower limbs.

## Methods

### Sample

All participants in this study were members of the GAZEL cohort (n = 20 625; 15 010 are men), all employed by the French national power utility (EDF-GDF). [19] Each January, participants receive general questionnaires about lifestyle, health, and occupational status; in 2006 and 2012, questions about pain were included. Few subjects are lost to follow-up, although not all subjects answer the questionnaire every year. The present analysis included men who answered both 2006 and 2012 questionnaires (n = 9 450). For each analysis, we excluded those reporting severe pain in 2006 (n = 1 443 for shoulder, n = 1 408 for knee), to determine the number of new cases (incident cases) that developed by 2012. We also excluded underweight participants (n = 35) and those missing 2006 data on smoking (n = 416), and BMI (n = 246). Thus, our final analytic n = 7310 for shoulder pain and n = 7345 for knee pain. We excluded women because of low prevalence of biomechanical exposures (4.82% exposed to elevated arms, 3.15% to squatting).

### Variables

The main outcome variables in this study are severe shoulder and severe knee pain in 2012. Pain was reported on a scale of 1 (lowest pain) to 8 (highest pain). We dichotomized the scale at the midpoint (severe pain ≥5, little to no pain ≤4) based on French convention. [5], [6], [8] Our main exposure variable was lifetime exposure to each of eight physical occupational tasks, retrospectively self-reported in 2006. Participants were asked for how long (never, <10 years, ≥10 years) they were exposed to “working with one or two arms in the air (above the shoulders) regularly or in a prolonged manner” (for shoulder pain analyses) or “working in a squatting position” (for knee pain analyses). BMI (kg/m^2^) in 2006, using self-reported height and weight, was categorized as normal (≥18.5–<25 kg/m^2^, overweight (≥25–<30 kg/m^2^), or obese (≥30 kg/m^2^). We also included age and current smoking in 2006 (yes/no).

### Analysis

We determined the number of incident cases in 2012 by excluding those with severe pain in 2006, and counting only new cases. We modeled associations between occupational factors, BMI, and new shoulder or knee pain in 2012 using logistic regression, estimating odds ratios (OR) and confidence intervals (95% CI). We present results stratified by BMI categories to illustrate the modifying effect of BMI on relationships between occupational factors and pain. Multiplicative interactions were also tested between BMI and occupational factors. All models were adjusted for age and smoking. Stata/MP, version 12.1, was used for all statistical analyses (StataCorp LP, College Station, TX, USA). Associations were considered statistically significant if two-tailed P-values were <0.05.

Authorization from the appropriate ethics committee was obtained (« Comité Consultatif National d’Ethique pour les Sciences de la Vie et de la Santé »; « Commission nationale de l’informatique et des libertés »).

## Results

A total of 8 753 men were included, aged 63–73 years old in 2012. Mean (SD) BMI was 26.59 kg/m^2^ +/−3.5 in 2012.

First, we examined associations between long-term (≥10 years) elevated arms, BMI, and severe shoulder pain. Elevated arm exposure was associated with increased risk of severe shoulder pain (OR 1.33, 95% CI [1.13,1.57] for <10 years, OR 1.61, [1.32,1.97] ≥10 years). When we added BMI, effects of occupational exposure were minimally attenuated to OR 1.31 [1.12,1.55] for <10 years of exposure, and attenuated to OR 1.54 [1.26,1.90] for ≥10 years of exposure. (Table 1). Obesity is also an independent risk factor for shoulder pain; obesity in 2006 associated with 2012 incidence of severe shoulder pain (OR 1.28 [1.03,1.90]). Multiplicative interaction between elevated arms and BMI on severe shoulder pain was non-significant. However, because the interaction between squatting and BMI was statistically significant for knee pain (p = 0.019), we present stratified analyses of both knee and shoulder pain in Table 2.

**Table 1:** Association between severe musculoskeletal pain (knee and shoulder) and relevant occupational exposure among men without severe pain in the related region in 2006.

	Numbers of subjects, cases and %			Adjusted Analyses excluding BMI		Adjusted Analyses Including BMI	
	Shoulder Pain in 2012^a^						
Duration of occupational factors (elevated arms)	Total (N)	Number of cases (n)	% Cases	OR c	95% CI	OR d	95% CI
Never	5013	512	10.21	1	-	1	-
<10 years	1866	250	13.4	1.33	1.13, 1.57	1.31	1.12, 1.55
≥10 years	978	149	15.24	1.61	1.32, 1.97	1.54	1.26, 1.90
BMI							
Normal	2762	306	11.08	-	-	1	-
Overweight	3841	433	11.27	-	-	0.97	0.83, 1.14
Obese	1032	147	14.24	-	-	1.28	1.03, 1.90
	**Knee Pain in 2012** b						
**Duration of occupational factors (squatting)**	**Total (N)**	**Number of** **cases (n)**	**n/N (%)**	**OR** c	**95% CI**	**OR** d	**95% CI**
Never	4707	437	9.28	1		1	
<10 years	1938	212	10.94	1.23	1.03, 1.46	1.91	1.23,2.98
≥10 years	1259	165	13.11	1.48	1.22,1.81	3.78	1.64,8.72
BMI							
Normal	2811	232	8.25	-	-	1	-
Overweight	3885	418	10.76	-	-	1.71	1.28, 2.29
Obese	986	139	14.10	-	-	3.21	1.90, 5.41
Interaction Term						0.019	0.73, 0.97

% = proportion; OR = odds ratio; 95% CI = 95% confidence interval.

a excluding those with shoulder pain in 2006,

b excluding those with knee pain in 2006,

c adjusted on age and smoking status,

d adjusted on BMI, gender, age and smoking status.

**Table 2:** Associations between occupational exposures and musculoskeletal pain, stratified on categories of body mass index (BMI).

	Shoulder Pain 2012 andnormal weight in 2006^a^					Shoulder Pain in 2012 andoverweight in 2006^a^					Shoulder Pain 2012 andobese in 2006^a^				
Duration ofelevated arms	Total(N)	Numberof cases(n)	%Cases	OR^c^	95%CI	Total(N)	Numberof cases(n)	%Cases	OR^c^	95%CI	Total(N)	Numberof cases(n)	%Cases	OR^c^	95%CI
Never	1885	182	9.66	1		2399	245	10.21	1		577	72	12.48	1	
<10 years	612	83	13.56	1.42	1.07, 1.89	940	115	12.23	1.2	.94, 1.52	277	47	16.97	1.41	.94, 2.12
≥10 years	265	41	15.47	1.7	1.17, 2.47	502	73	14.54	1.54	1.15, 2.04	178	28	15.73	1.34	.83, 2.15
	**Knee Pain 2012 and** **normal weight in 2006** b					**Knee Pain in 2012 and** **overweight in 2006** b					**Knee Pain 2012 and obese** **in 2006** b				
**Duration** **of** **squatting**	**Total** **(N)**	**Number** **of cases** **(n)**	**%** **Cases**	**OR** c	**95%** **CI**	**Total (N)**	**Number** **of cases** **(n)**	**%** **Cases**	**OR** c	**95% CI**	**Total** **(N)**	**Number** **of cases** **(n)**	**%** **Cases**	**OR** c	**95%** **CI**
Never	1778	126	7.09	1		2273	225	9.9	1		517	74	14.31	1	
<10 years	642	54	8.41	1.21	.86, 1.70	987	113	11.45	1.22	.96, 1.56	264	37	14.02	0.98	.63, 1.52
≥10 years	391	52	13.3	2.03	1.42, 2.88	625	80	12.8	1.32	.99, 1.76	205	28	13.66	0.99	.61, 1.58

% = proportion; OR = odds ratio; 95% CI = 95% confidence interval.

a excluding those with shoulder pain in 2006,

b excluding those with knee pain in 2006,

c adjusted on age and smoking status.

We next tested relationships between long-term squatting exposure, BMI, and knee pain. We found that squatting was associated with increased risk of severe knee pain (adjusted OR 1.23, 95% CI [1.03,1.46] for <10 years, OR 1.48 [1.22,1.81] ≥10 years). When we added BMI, effects of occupational exposures increased to OR 1.91 [CI 1.23,2.98] for <10 years of exposure, and increased to OR 3.78 [1.64,8.72] for ≥10 years of exposure (Table 1).

Overweight and obesity were also independent risk factors for knee pain; compared with normal-weight individuals, overweight was significantly associated with the incidence of severe knee pain in 2012 (OR 1.71 [1.28,2.29]), as was obesity in 2006 (OR 3.21 [1.90,5.41]). In stratified results for knee pain in 2012, exposure to squatting for ≥10 years in normal-weight individuals significantly predicts knee pain (OR 2.03 [1.42, 2.88]). However in obese and overweight participants, occupational exposure for >10 years is not statistically significantly associated with knee pain (overweight: OR 1.32 [0.99,1.76]; obese: OR 0.99 [0.61,1.58]). For occupational exposure of <10 years, stratified BMI is not significantly associated with knee pain.

## Discussion

This study found a complex interplay among overweight and obese patients’ long-term occupational physical exposures and severe pain in the shoulders and knees. As in previous studies, we found that elevated arms or squatting increases risk for shoulder and knee pain respectively, [1]–[4], [10], that obesity is a significant risk factor for shoulder pain, and that overweight/obesity are significant risk factors for knee pain. [10], [11] However, this study is novel in that we stratified by BMI, further dissecting the relationship between obesity, occupational exposures, and musculoskeletal pain.

We found that, for knee pain, the interaction between BMI and the occupational factor, squatting, was significant. Subjects of normal BMI have increased risk of knee pain when exposed to squatting for greater than 10 years, but obese subjects do not. This is interesting because one would expect obese patients to have an increased risk of knee pain over patients of a normal BMI, due to increased weight and pressure on the knees. One theory is that obese individuals are placed in positions with minimal squatting compared to normal-weight individuals. Of note, new cases of severe shoulder and knee pain seen in our cohort occurred after retirement, demonstrating prolonged negative health effects of obesity and work exposures after exposure cessation.

This study has several limitations. Retirement age in GAZEL is relatively young (55 years), though this should have minimal effects on associations between risk factors and outcomes. [5], [6] We studied men only, although some studies have shown no difference between men and women in this area. [16] Occupational exposures were self-reported, though six years before the outcome measures, making biased reporting of exposure with respect to outcome unlikely. Exposure data (occupational and weight measurements) were relatively crude, though resultant random error likely biases results toward the null. It is outside of the scope of our study to describe trajectories of weight changes and occupational exposures during working years. It is conceivable, for instance, that workers’ jobs became more sedentary as obesity developed, which might explain lack of associations between occupational exposures and severe pain in obese patients. Finally, this preliminary study did not examine other possible risk factors for MSDs, such as diabetes, other medical conditions, physical activity, or socioeconomic status, which may influence associations between occupational factors and BMI and explain some associations (between BMI and exposure for instance). Some strengths of this study are the large sample size and characteristics of GAZEL, including high retention and survey response rates.

This study provides insight on the nature of relationships between occupational factors, obesity, and musculoskeletal pain. We found that, after adjusting for occupational factors, high BMI was associated with lower and upper limb pain, as was also shown in a recently published study that found that BMI was associated with musculoskeletal symptoms. [18] Our study builds on that finding by also considering the role of occupational exposures in this relationship. Although causality cannot be inferred, our findings suggest that obesity might be a moderating factor, as suggested for osteoarthritis recently. [13] Results for obese workers should be interpreted with caution, as such individuals may be placed in jobs that require less squatting, thus lowering risk for musculoskeletal symptoms.

From a practical perspective, these results suggest that musculoskeletal pain among obese and overweight workers might also be a result of working conditions (at least for the shoulder) and should be taken into account by physicians as such. From a research perspective, studies of associations between occupational exposures and specific MSDs would benefit from inclusion of metrics such as levels of obesity or adiposity in order to better explain observed associations.

## References

[1] Punnett L, Wegman DH (2004) Work-related musculoskeletal disorders: the epidemiologic evidence and the debate. J Electromyogr Kinesiol Off J Int Soc Electrophysiol Kinesiol 14: 13–23 10.1016/j.jelekin.2003.09.015 14759746

[2] Van Rijn RM, Huisstede BM, Koes BW, Burdorf A (2010) Associations between work-related factors and specific disorders of the shoulder–a systematic review of the literature. Scand J Work Environ Health 36: 189–201. 20094690 10.5271/sjweh.2895

[3] Miranda H, Viikari-Juntura E, Martikainen R, Riihimaki H (2002) A prospective study on knee pain and its risk factors. OsteoarthritisCartilage 10: 623–630. 10.1053/joca.2002.0796 12479384

[4] McWilliams DF, Leeb BF, Muthuri SG, Doherty M, Zhang W (2011) Occupational risk factors for osteoarthritis of the knee: a meta-analysis. Osteoarthr Cartil OARS Osteoarthr Res Soc 19: 829–839 10.1016/j.joca.2011.02.016 21382500

[5] Descatha A, Cyr D, Imbernon E, Chastang J-F, Plenet A, et al (2011) Long-term effects of biomechanical exposure on severe knee pain in the Gazel cohort. Scand J Work Environ Health 37: 37–44. 20886201 10.5271/sjweh.3123 PMC3124467

[6] Descatha A, Teysseyre D, Cyr D, Imbernon E, Chastang J-F, et al (2012) Long-term effects of biomechanical exposure on severe shoulder pain in the Gazel cohort. Scand J Work Environ Health 38: 568–576 10.5271/sjweh.3300 22527281

[7] Kuorinka I, Jonsson B, Kilbom A, Vinterberg H, Biering-Sorensen F, et al (1987) Standardised Nordic questionnaires for the analysis of musculoskeletal symptoms. ApplErgon 18: 233–237. 10.1016/0003-6870(87)90010-x 15676628

[8] Hagberg M, Violante F, Bonfiglioli R, Descatha A, Gold J, et al (2012) Prevention of musculoskeletal disorders in workers: classification and health surveillance - statements of the Scientific Committee on Musculoskeletal Disorders of the International Commission on Occupational Health. BMC Musculoskelet Disord 13: 109 10.1186/1471-2474-13-109 22721454 PMC3437218

[9] Charles M-A, Eschwège E, Basdevant A (2008) Monitoring the obesity epidemic in France: the Obepi surveys 1997–2006. Obes Silver Spring Md 16: 2182–2186 10.1038/oby.2008.285 PMC2665194 18535547

[10] Anderson JJ, Felson DT (1988) Factors associated with osteoarthritis of the knee in the first national Health and Nutrition Examination Survey (HANES I). Evidence for an association with overweight, race, and physical demands of work. Am J Epidemiol 128: 179–189. 3381825 10.1093/oxfordjournals.aje.a114939

[11] Wearing SC, Hennig EM, Byrne NM, Steele JR, Hills AP (2006) Musculoskeletal disorders associated with obesity: a biomechanical perspective. Obes Rev Off J Int Assoc Study Obes 7: 239–250 10.1111/j.1467-789X.2006.00251.x 16866972

[12] Coggon D, Reading I, Croft P, McLaren M, Barrett D, et al (2001) Knee osteoarthritis and obesity. Int J ObesRelat Metab Disord 25: 622–627. 10.1038/sj.ijo.0801585 11360143

[13] Martin KR, Kuh D, Harris TB, Guralnik JM, Coggon D, et al (2013) Body mass index, occupational activity, and leisure-time physical activity: an exploration of risk factors and modifiers for knee osteoarthritis in the 1946 British birth cohort. BMC Musculoskelet Disord 14: 219 10.1186/1471-2474-14-219 23883324 PMC3726290

[14] Murray CJL, Vos T, Lozano R, Naghavi M, Flaxman AD, et al (2012) Disability-adjusted life years (DALYs) for 291 diseases and injuries in 21 regions, 1990–2010: a systematic analysis for the Global Burden of Disease Study 2010. The Lancet 380: 2197–2223 10.1016/S0140-6736(12)61689-4 23245608

[15] Pandalai SP, Schulte PA, Miller DB (2013) Conceptual heuristic models of the interrelationships between obesity and the occupational environment. Scand J Work Environ Health. doi:10.5271/sjweh.3363. 10.5271/sjweh.3363 PMC4623304 23588858

[16] Bonde JPE, Viikari-Juntura E (2013) The obesity epidemic in the occupational health context. Scand J Work Environ Health 39: 217–219 10.5271/sjweh.3362 23588821

[17] Luckhaupt SE, Cohen MA, Li J, Calvert GM (2014) Prevalence of obesity among U.S. workers and associations with occupational factors. Am J Prev Med 46: 237–248 10.1016/j.amepre.2013.11.002 24512862

[18] Viester L, Verhagen EALM, Oude Hengel KM, Koppes LLJ, van der Beek AJ, et al (2013) The relation between body mass index and musculoskeletal symptoms in the working population. BMC Musculoskelet Disord 14: 238 10.1186/1471-2474-14-238 23937768 PMC3751130

[19] Goldberg M, Leclerc A, Bonenfant S, Chastang JF, Schmaus A, et al (2007) Cohort profile: the GAZEL Cohort Study. IntJ Epidemiol 36: 32–39. 17101614 10.1093/ije/dyl247 PMC2258334

